# Central nervous system plasmablastic lymphoma evolving from polyclonal plasmacytosis

**DOI:** 10.1111/bjh.15489

**Published:** 2018-07-09

**Authors:** Umair T. Khan, Igor Racu‐Amoasii, Arvind Arumainathan, Ushma Meswani, Andrew R. Pettitt, Geetha Menon

**Affiliations:** ^1^ Department of Haematology Clatterbridge Cancer Centre Liverpool UK; ^2^ Institute of Translational Medicine Department of Molecular and Clinical Cancer Medicine University of Liverpool Liverpool UK; ^3^ Haemato‐oncology Diagnostic Service Liverpool Clinical Laboratories Liverpool UK; ^4^ Department of Haematology Countess of Chester Hospitals NHS Trust Chester UK

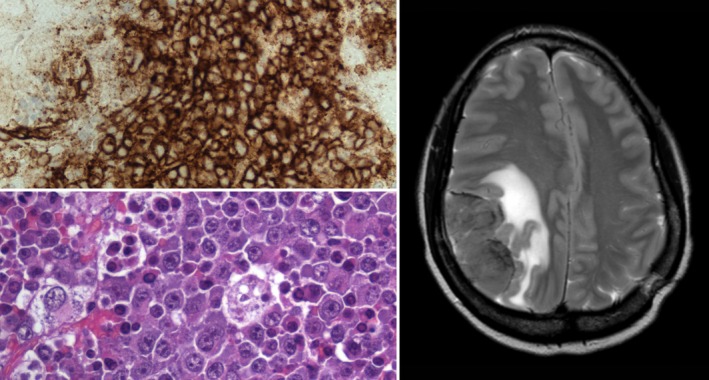

A 29‐year‐old woman presented with severe headaches, vomiting and drowsiness in January 2016. A computed tomography scan of the brain at the time showed bilateral subdural haematomas with midline shift, sub‐falcine herniation and hydrocephalus. The patient underwent a burr‐hole procedure with surgical evacuation of the haematomas. At the time of surgery, an extradural mass was noted and biopsied.

Further investigation showed that the patient was human immunodeficiency virus (HIV) seropositive with an Epstein–Barr virus (EBV) viraemia of 50 400 copies/ml and a cytomegalovirus viraemia of <250 copies/ml by polymerase chain reaction. She was noted to have polyclonal hypergammaglobulinaemia with a serum immunoglobulin G level of 41·2 g/l. Screening for tuberculosis, schistosomiasis and human herpesvirus 8 viraemia was negative. The histology from the extradural mass showed an EBV‐associated polyclonal expansion of plasma cells with no evidence of lymphoma (top left, CD138 immunohistochemical stain).

The patient commenced anti‐retroviral therapy in the expectation that the EBV‐associated plasma cell proliferation would regress following restoration of immune function in the absence of systemic lymphoma. However, the patient re‐presented in April 2016 with severe headache and left hemiparesis. Magnetic resonance imaging at that time showed a new 6·6 × 3·2 × 5 cm enhancing extra‐axial mass lesion in the right parietal region (right), which, on repeat biopsy showed plasmablastic lymphoma (bottom left). The malignant cells in this case had a plasma cell phenotype, with expression of CD10 and CD138, and were strongly positive for EBV‐EBER (EBV‐encoded early ribonucleic acid). The patient commenced the MATRix regimen (methotrexate, cytarabine, thiotepa, rituximab) after pre‐treatment with dexamethasone but failed to respond after 2 cycles and went on to receive palliative radiotherapy. She subsequently developed bowel obstruction and died in February 2017.

We describe an EBV‐associated polyclonal expansion of plasma cells occurring in the brain from which a primary central nervous system (CNS) plasmablastic lymphoma apparently evolved. Primary CNS plasmablastic lymphoma is very rare, with only eight cases reported in the literature. EBV‐associated polyclonal expansion of plasma cells (with the propensity to evolve into lymphoma) is a well‐known complication of allogeneic haemopoietic stem cell transplantation but is less well‐described in the context of HIV infection. In the latter setting, plasmablastic lymphoma usually occurs *de novo* and predominantly affects the oral cavity, upper aerodigestive tract, orbit, skin, bone, soft tissues and gastrointestinal tract. The clinical course of our patient is in keeping with the dismal prognosis of plasmablastic lymphoma, with most patients dying within a year of diagnosis.

